# Metabolomic Profiles of Bovine Mammary Epithelial Cells Stimulated by Lipopolysaccharide

**DOI:** 10.1038/s41598-019-55556-2

**Published:** 2019-12-13

**Authors:** Yixin Huang, Liuhong Shen, Jing Jiang, Qipin Xu, Zhengzhong Luo, Qiao Luo, Shumin Yu, Xueping Yao, Zhihua Ren, Yanchun Hu, Yongxin Yang, Suizhong Cao

**Affiliations:** 1 Department of Clinical Veterinary Medicine, College of Veterinary Medicine, Sichuan Agricultural University, Chengdu, 611130 China; 2 Sichuan Provincial Key Laboratory of Animal Diseases and Human Health, Chengdu, 611130 China; 3 Institute of Biodiversity Animal Health & Comparative Medicine, College of Medical, Veterinary & Life Sciences, University of Glasgow, Glasgow, G61 1QH UK; 4 Institute of Animal Science and Veterinary Medicine, Anhui Academy of Agricultural Sciences, Hefei, 230031 China

**Keywords:** Metabolomics, Mass spectrometry

## Abstract

Bovine mammary epithelial cells (bMECs) are the main cells of the dairy cow mammary gland. In addition to their role in milk production, they are effector cells of mammary immunity. However, there is little information about changes in metabolites of bMECs when stimulated by lipopolysaccharide (LPS). This study describes a metabolomics analysis of the LPS-stimulated bMECs to provide a basis for the identification of potential diagnostic screening biomarkers and possible treatments for bovine mammary gland inflammation. In the present study, bMECs were challenged with 500 ng/mL LPS and samples were taken at 0 h, 12 h and 24 h post stimulation. Metabolic changes were investigated using high performance liquid chromatography-quadrupole time-of-flight mass spectrometry (HPLC-Q-TOF MS) with univariate and multivariate statistical analyses. Clustering and metabolic pathway changes were established by MetaboAnalyst. Sixty-three differential metabolites were identified, including glycerophosphocholine, glycerol-3-phosphate, L-carnitine, L-aspartate, glutathione, prostaglandin G2, α-linolenic acid and linoleic acid. They were mainly involved in eight pathways, including D-glutamine and D-glutamic acid metabolism; linoleic acid metabolism; α-linolenic metabolism; and phospholipid metabolism. The results suggest that bMECs are able to regulate pro-inflammatory, anti-inflammatory, antioxidation and energy-producing related metabolites through lipid, antioxidation and energy metabolism in response to inflammatory stimuli.

## Introduction

Bovine mastitis is common and is economically one of the most important diseases that seriously affects the health and welfare of dairy cows. It is an inflammatory condition of the mammary gland, reducing milk quality and production, impairing fertility, causing mortality^1,2^, and is a food-borne disease that increases human public health risks^3^.

Bovine mastitis is commonly caused by bacteria including *Staphylococcus aureus*, *Escherichia coli* and *Streptococcus*^2,3^. LPS, the major component of the outer membrane of gram-negative bacteria, is a bacterial virulence factors that can induce strong immune responses in animals. In mammary gland, bMECs are important milk-producing cells and key regulators of immunity^3–5^. Research on bMECs is not only conducive to the understanding of growth and development of mammary gland and regulation of lactation, but also to a deeper understanding of mammary immunity. Thus LPS-stimulated bMECs have been widely used in studying bMECs immune response mechanisms or bovine mastitis.

Studies have shown that simulation of bMECs with LPS can generate strong immune responses, upregulating the expression of pathogen-associated molecular patterns (PAMPs), activating nuclear factor-κB (NF-κB) signaling pathway, and increasing the secretion of inflammatory cytokines^3,5^. In a study of bMECs^4^, the mRNA expression levels of Toll-like receptor 4 (*TLR4*) and *TLR2* were significantly increased by stimulation with 0.01 μg/mL, 1 μg/mL, 5 μg/mL and 10 μg/mL LPS for 24 h when compared to controls (without LPS). Significant upregulation of *TLR4* or *TLR2* mRNA expression was observed as early as 6 h (*p* < 0.05) or 24 h (*p* < 0.01) respectively after stimulation of 1 μg/mL LPS. Wu *et al*.^5^ found that after bMECs were stimulated with 10 μg/mL LPS for 12 h, the gene and protein expression levels of TLR4, NF-κB p65 and NF-κB p65 phosphorylation were significantly increased (*p* < 0.05). The mRNA expression of interleukin-1β (*IL-1β*), *IL-6*, tumor necrosis factor-α (*TNF-α*), inducible nitric oxide synthase (*iNOS*) and *NO* content increased significantly (*p* < 0.05) as well. After 10 ng/mL LPS stimulation for 24 h in bMECs^1^, mRNA transcription of the enzymes responsible for arachidonic acid (AA) metabolism increased significantly, including prostaglandin (PG)-F synthase (*p* < 0.001), prostaglandin-endoperoxide synthase 2 and PGE synthase (*p* < 0.01). PGF_2α_ (*p* < 0.01), leukotriene -B_4_ (*p* < 0.05) and LTC_4_ (*p* < 0.001) secretion in bMECs were all increased. Differentially expressed proteins that were associated with inflammatory model of bMECs induced by LPS were screened by isobaric tag for relative and absolute quantification (iTRAQ), combined with 2-dimensional liquid chromatography- tandem mass spectrometry (2D-LC-MS/MS), indicating that LPS treatment could affect the synthesis of protein and fat in bMECs^6^. However, there is little information about changes in metabolite levels of bMECs when they are stimulated by LPS.

In this study, our aim was to identify potential biomarkers for the diagnosis and treatment of bovine mammary gland inflammation. Previous studies showed that pro-inflammatory cytokines usually promote inflammation quickly after the recognition of the pathogen, and then anti-inflammatory cytokines suppress and limit their activity^3^. The expression of mRNA and proteins of cytokines including IL-6, TNF-α, and enzymes including cyclooxygenase (COX) - 2, lipoxygenase (LOX) - 5 were measured in a pilot study, which suggested that 500 ng/mL of LPS and two time-points (12 h and 24 h after LPS stimulation) would be suitable for our study. We then used HPLC-Q-TOF MS technique, with the aim of identifying differentially produced metabolites and the relevant pathways of the inflammatory response in bMECs after LPS stimulation.

## Results

### The mRNA and protein expression of cytokines and enzymes

**Figure 1:**
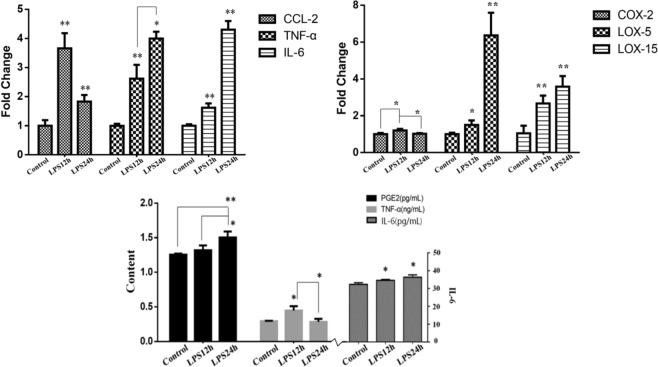
The mRNA (RT-qPCR) and protein (ELISA) expression of cytokines and enzymes. *Means *p* < 0.05, **means *p* < 0.01 (n = 3). It represents that the treatment group is compared with Control if there is no special indication.

To determine the inflammatory conditions of bMECs, changes of several genes and proteins were investigated (Fig. 1). We found that the mRNA expression of chemokine (c-c motif) ligand (*CCL)-2*, *IL-6* and *TNF-α* in LPS12h increased significantly (*p* < 0.01) compared to Control. When compared LPS24h with LPS12h, the mRNA expression of *IL-6* and *TNF-α* increased, while *CCL-2* decreased but it was still higher than that in Control (*p* < 0.01). In LPS12h, the mRNA expression of enzymes *COX-2*, *LOX-5* and *LOX-15* were up-regulated significantly when compared with Control. In LPS24h, the mRNA expression of *COX-2* decreased significantly when compared with LPS12h. The mRNA expression of *LOX-5* and *LOX-15* increased significantly (*p* < 0.01) when compared with Control. The results of protein expression showed that the secretion of IL-6, TNF-α and PGE2 increased in LPS12h compared to Control. When compared LPS24h with Control, the secretion of PGE2 and IL-6 were up-regulated significantly. There was a significant drop of TNF-α in LPS24h compared to LPS12h. These results indicated that 500 ng/mL of LPS and two time-points (12 h and 24 h after LPS stimulation) were suitable for our study to establish an inflammatory model of bMECs.

### Metabolic profiles

**Figure 2:**
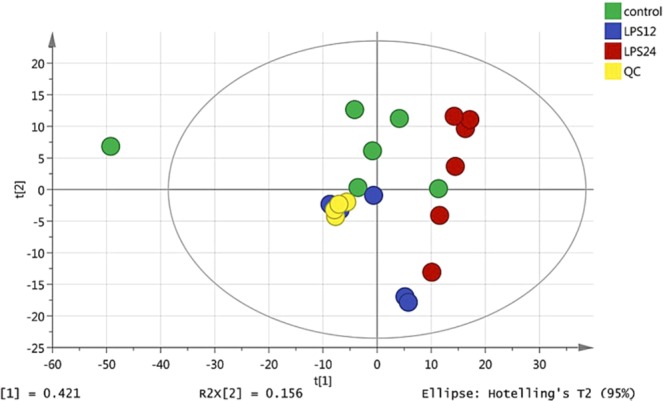
PCA score plots of three groups (with QC).

**Figure 3:**
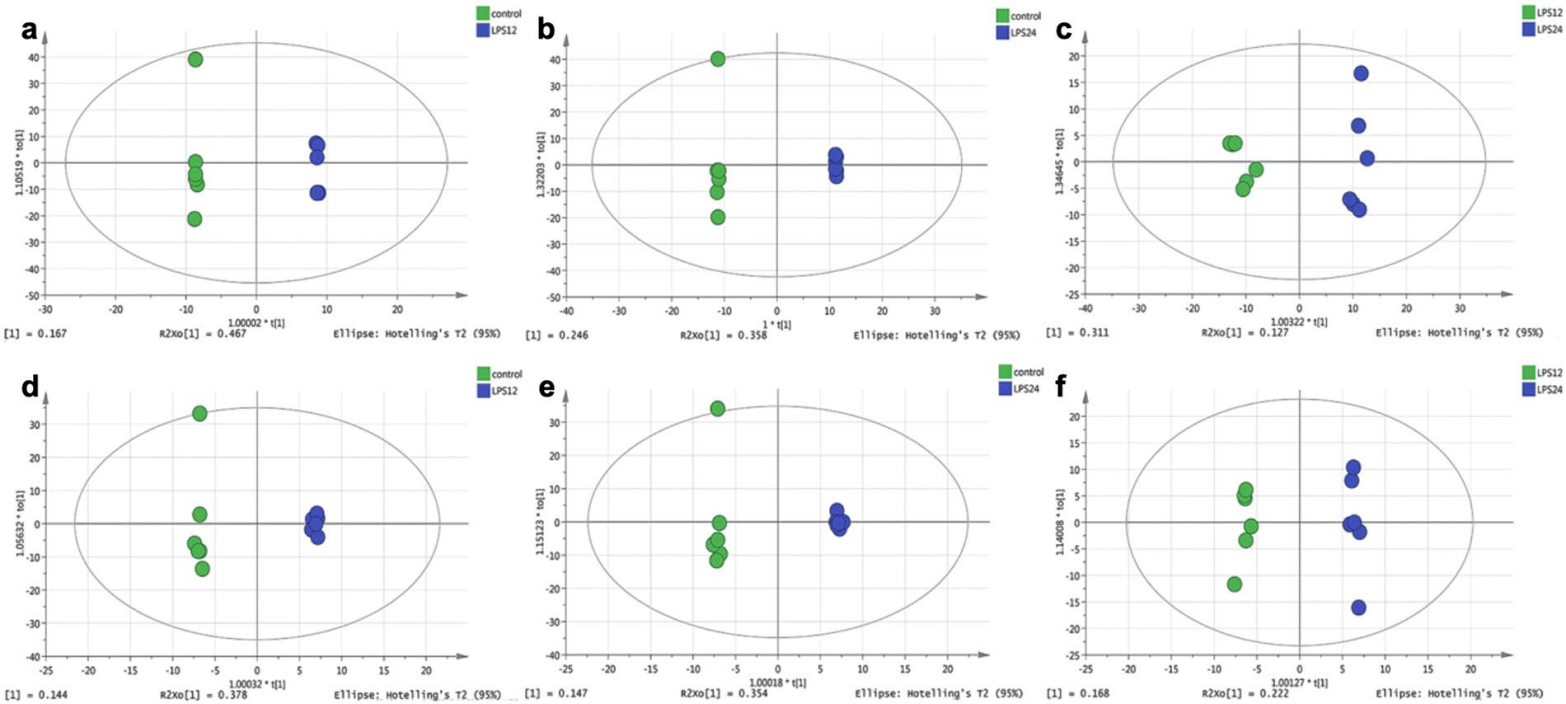
OPLS-DA score plots of all groups. (**a–c**) Are the OPLS-DA score plots between LPS12h and Control, LPS24h and Control, LPS12h and LPS24h in ESI+, respectively; (**d–f**) are the OPLS-DA score plots between LPS12h and Control, LPS24h and Control, LPS12h and LPS24h in ESI-, respectively.

Representative HPLC-Q-TOF MS electrospray ionization (ESI) ± chromatograms are shown in Supplementary Fig. S1. 3112 peaks and 4773 peaks were extracted in ESI- and ESI + , respectively. Figure 2 displays the similarities and differences among Control (green circles), LPS12h (blue circles) and LPS24h (red circles) in the score plots of PCA (principal component analysis). The quality control (QC) samples (yellow circles) were tightly clustered, indicating that the method used was robust, with high repeatability and stability. Supplementary Fig. S2 shows the results of partial least squares discriminant analysis (PLS-DA), in which the samples were aggregated within treatments, which were mostly distinct from each other. Table 1 and Fig. 3 are orthogonal partial least squares discriminate analysis (OPLS-DA) results, indicating that the model was stable and reliable, with good predictability.

**Table 1:** Evaluation Parameters of OPLS-DA models.

Groups	ESI+		ESI−	
	R2Y	Q2	R2Y	Q2
LPS12h-Control	1	0.883	0.999	0.826
LPS24h-Control	1	0.943	0.999	0.789
LPS24h-LPS12h	0.984	0.787	0.994	0.64

R2Y represents the rate of model interpretation, and Q2 represents the model predictive ability.

### Discovery and identification of the differential metabolites

**Figure 4:**
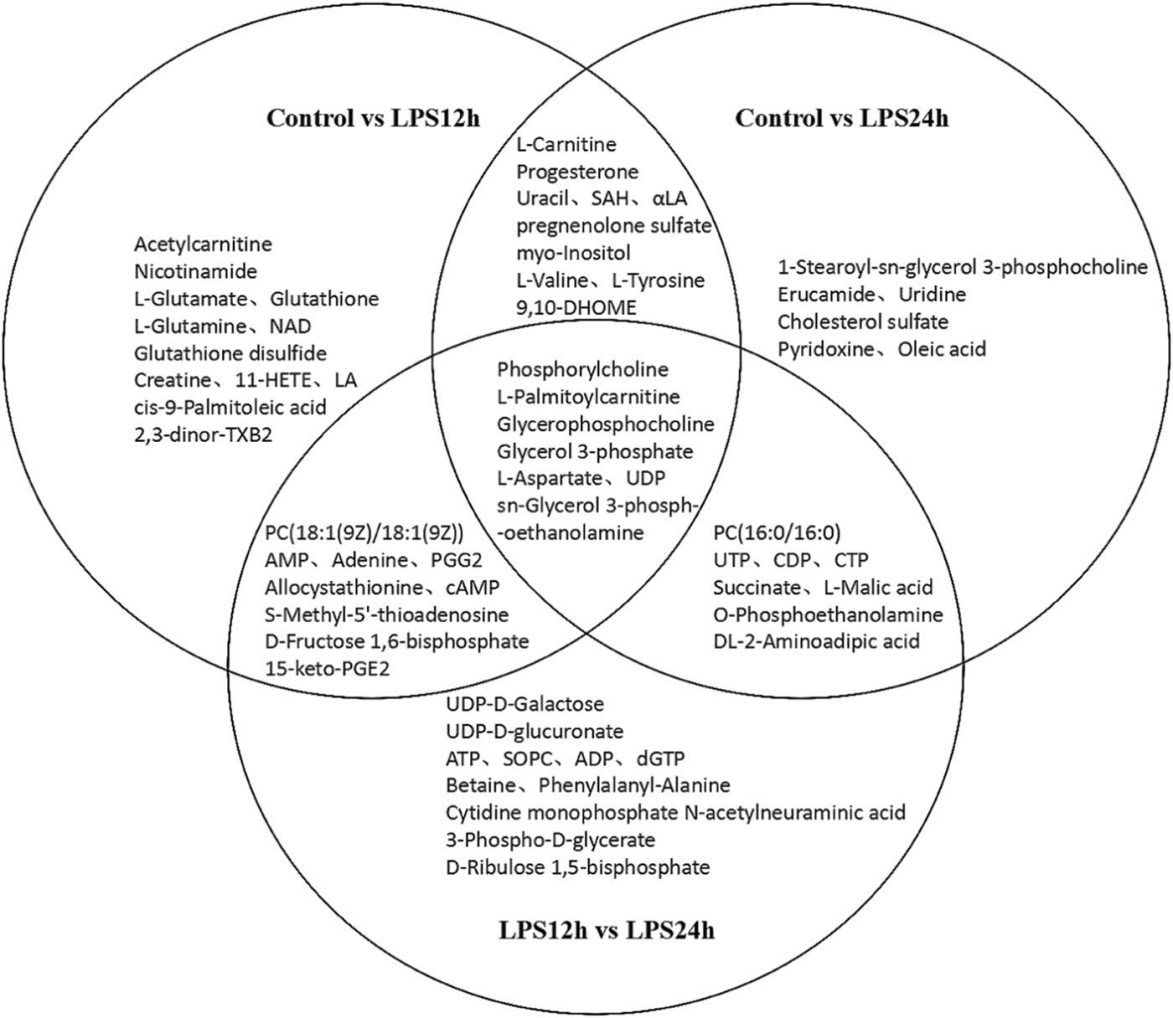
Detection of significant differential metabolites in different groups. NAD: Nicotinamide-adenine dinucleotide; 11HETE: 11-Hydroxyeicosatetraenoic acid; LA: Linoleic acid; 2,3-dinor-TXB2: 2,3-dinor-thromboxane B2; PC: Phosphatidylcholine; PGG2: Prostaglandin G2; cAMP: cyclic Adenosine monophosphate; 15-keto-PGE2: 15-keto Prostaglandin E2; SAH: S-Adenosylhomocysteine; αLA: alpha-Linolenic acid; 9,10-DHOME: 9,10-dihydroxy-12Z-octadecenoic acid; UDP: Uridine diphosphate; UTP: Uridine triphosphate; CDP: Cytidine diphosphate; CTP: Cytidine triphosphate; ATP: Adenosine triphosphate; SOPC: 1-stearoyl-2-oleoyl-sn-glycerol-phosphocholine; ADP: Adenosine diphosphate; dGTP: Deoxyguanosine triphosphate.

According to the variable importance in projection (VIP) value obtained by OPLS-DA and the results of *t*-tests, metabolites that had both VIP > 1 and *p* < 0.05 were selected as significantly differential metabolites. A total of 63 metabolites at significantly different abundance according to treatment were identified, including 18 nucleotides and their derivatives, 22 lipids, 13 organic acids and their derivatives, and 10 other organic compounds. Among them, 10 same metabolites changed significantly both in LPS12h and LPS24h when compared to Control; 10 same metabolites were found to be different in Control vs LPS12h and LPS24h vs LPS12h; 8 were found to be different both in Control vs LPS24h and LPS12h vs LPS24h; 7 same metabolites differed in all three treatment groups (Fig. 4).

**Figure 5:**
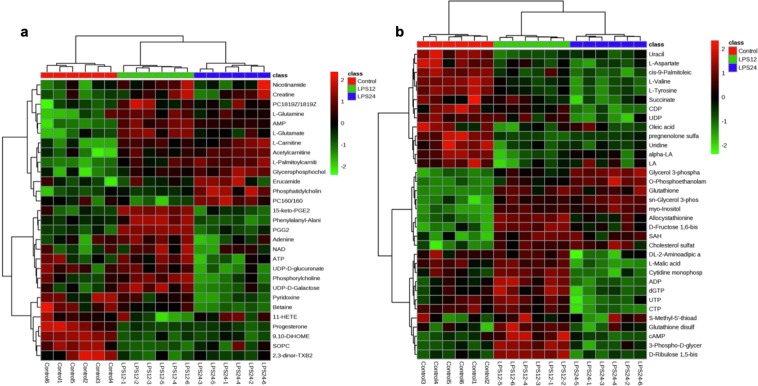
Hierarchical clustering of differential metabolites. (**a**) Hierarchical clustering of 29 differential metabolites in ESI+. (**b**) Hierarchical clustering of 34 differential metabolites in ESI−.

Hierarchical clustering of differential metabolites was performed by MetaboAnalyst 4.0 (Fig. 5). It showed that related metabolites and the treatment groups were clustered together. Some metabolites in bMECs in treatments LPS12h and LPS24h showed highly significant increases when compared with Control samples (*p* < 0.01), and were significantly up-regulated in LPS24h compared with LPS12h (*p* < 0.05). These included PC (18:1(9Z)/18:1(9Z)), sn-glycerol 3-phosphoethanolamine, glycerol 3-phosphate and glycerophosphocholine. L-aspartate was down-regulated in LPS12h and LPS24h compared to Control (*p* < 0.05) and showed a decrease in LPS24h compared to LPS12h as well (*p* < 0.05). Some metabolites, including PGG2 and 15-keto-PGE2 in LPS12h increased highly significantly when compared to Control (*p* < 0.01) but showed a highly significantly reduction in LPS24h when compared with LPS12h (*p* < 0.01). The metabolites α-linolenic acid (α-LA) and linoleic acid (LA) significantly decreased in LPS12h compared to Control (*p* < 0.05). More information of differential metabolites between groups are summarized in Supplementary Tables S1, S2 and S3.

### Characterization and functional analysis of the key metabolic pathways involved in bMECs inflammation

**Figure 6:**
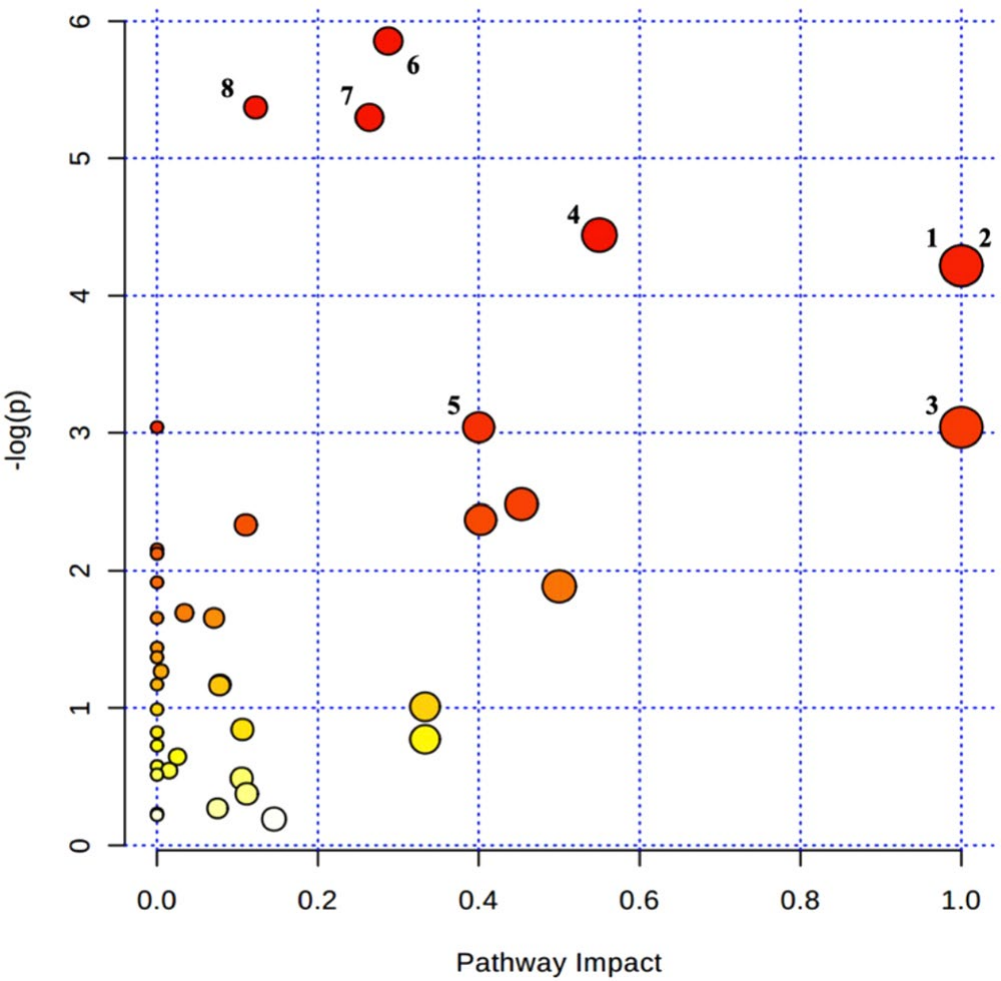
Metabolic pathway analysis. Circles represent metabolic pathways. Darker circles indicate more significant changes in the metabolites in the corresponding pathway, whereas the size of the circle corresponds to the Impact.

Based on the Impact and *p* values, 42 metabolic pathways in which potential biomarkers might be located were mapped by MetaboAnalyst (Fig. 6). The pathways in which both Impact >0.1 and *p* < 0.05 were considered as the highest potential metabolic pathways, among which 3 pathways had Impact of 1: D-glutamine and D-glutamic acid metabolism; Linoleic acid metabolism; and α -linolenic acid metabolism (Table 2).

**Table 2:** The Main metabolic pathways.

No.	Pathways	Impact	*p*
1	D-glutamine and D-glutamic acid metabolism	1	0.015
2	Linoleic acid metabolism	1	0.015
3	α -linolenic acid metabolism	1	0.048
4	Alanine, aspartate and glutamate metabolism	0.55	0.012
5	Ascorbate and aldarate metabolism	0.4	0.048
6	Pyrimidine metabolism	0.29	0.003
7	Phospholipid metabolism	0.26	0.005
8	Purine metabolism	0.12	0.005

Ordered by Impact from higher to lower.

**Figure 7:**
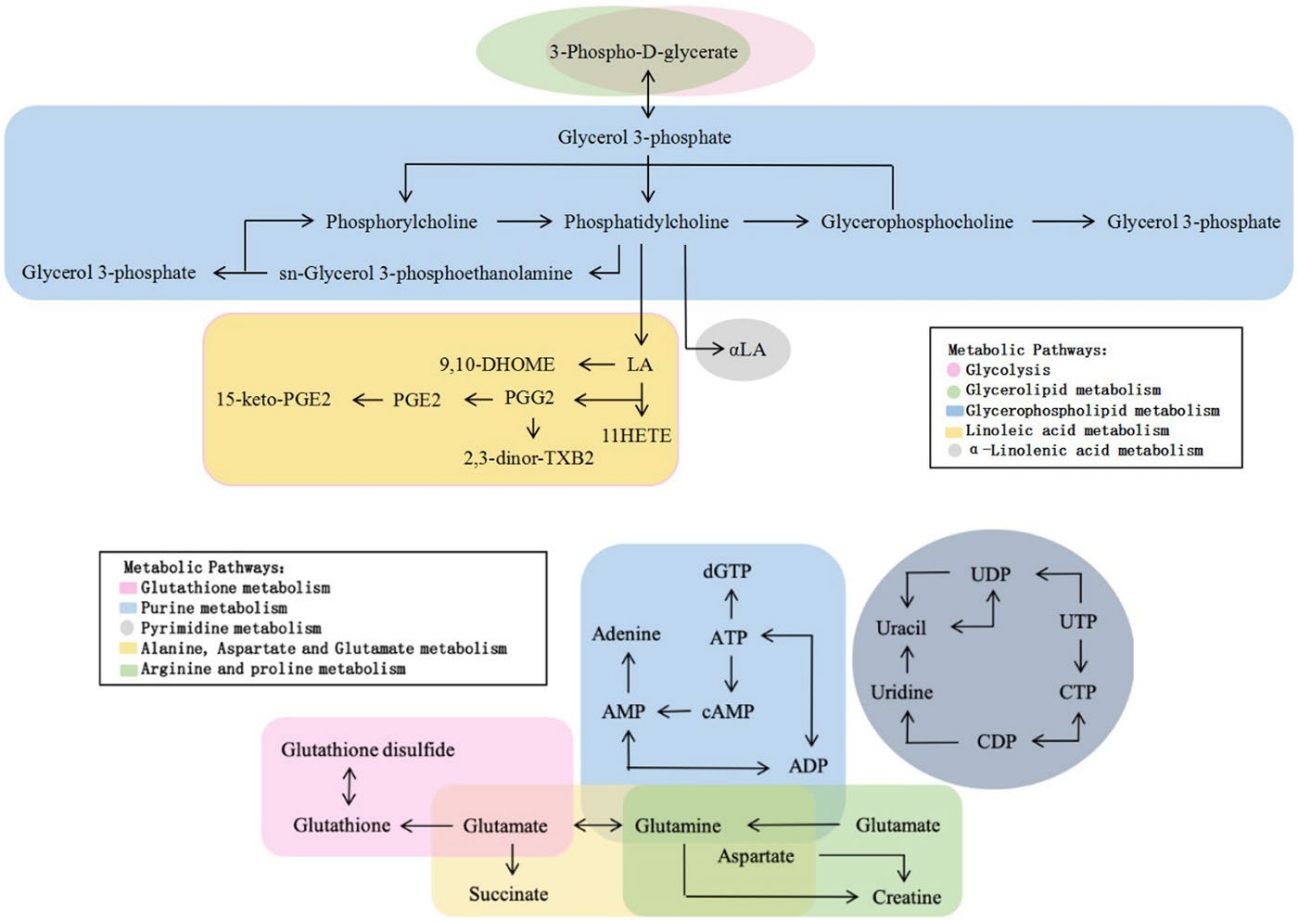
Interrelationship of the differential metabolites in the main metabolic pathways.

Interrelation maps of the differential metabolites in the main metabolic pathways were generated by analyzing Kyoto Encyclopedia of Genes and Genomes (KEGG) pathway database and publications (Fig. 7). The glycerophospholipid metabolic pathway can be associated with glycolysis and glycerolipid metabolic pathways via phosphoglycerate. Phosphatidylcholine in the glycerophospholipid metabolic pathway can generate α-LA and LA, thereby affecting α-LA and LA metabolic pathways. Glutamine is not only a metabolite in the purine metabolic pathway, but also participates in the metabolism of arginine and valine; as well as alanine, aspartic acid, and glutamate metabolism. Glutamate is involved in the formation of glutathione, and is not only closely related to glutamine, but also can be metabolized to succinate, participating the metabolism of alanine, aspartate and glutamate.

## Discussion

In the present study, there were 38 and 31 differential metabolites in LPS12h and LPS24h compared with Control, respectively, and 35 differential metabolites when LPS12h was compared with LPS24h. Pyrimidine metabolism; purine metabolism; phospholipid metabolism; alanine, aspartate and glutamate metabolism; D-glutamine and D-glutamic acid metabolism; linoleic acid metabolism; ascorbate and aldarate metabolism; and alpha-linolenic acid metabolism were found to be the main pathways that were involved, indicating that they were affected by LPS stimulation in bMECs.

After LPS stimulation there were significant changes in the metabolites that were involved in glycerophospholipid metabolism and glycerolipid metabolism. It was previously reported that glycerophospholipid metabolism changed significantly in the milk of mastitic cows when compared with healthy cows^7^. In that study, glycerophosphocholine, phosphorylcholine and phosphocholine decreased in the milk of cows with clinical mastitis. However, we found that glycerophosphocholine increased highly significantly in LPS12h and LPS24h (*p* < 0.01) when compared to Control. Phosphorylcholine significantly increased in LPS24h (*p* < 0.01) compared with Control, and it decreased when LPS24h was compared to LPS12h (*p* < 0.01). Phosphocholine, which is one of the intermediates in the synthesis of phosphatidylcholine (PC), was not found in our study. Studies have indicated that PCs, the main component of cell membrane, exhibit anti-inflammatory effects^8,9^. Thus the increases of 1-stearoyl-2-oleoyl-sn-glycerol-phosphocholine (18:0/18:1 PC, SOPC) (*p* < 0.05, LPS24h vs Control) and PC (18:1(9Z)/18:1(9Z)) (*p* < 0.05, LPS12h vs Control; *p* < 0.01, LPS24h vs LPS12h) suggest that they might have anti-inflammatory function in bMECs. Some studies have shown that PCs can inhibit lipid peroxidation to prevent cell membrane damage, by emulsifying and degrading fats, reducing the amount of neutral fat and removing peroxides^10^, which suggested that the PCs might also played an antioxidant role in bMECs in the present study. In contrast, lysophosphatidylcholines (LysoPCs, LPCs), the metabolites of PCs, have been reported to exhibit pro-inflammatory effects^9,11,12^. In the present study, 1-stearoyl-sn-glycero-3-phosphocholine, an LPC, showed a significantly increase in bMECs after LPS stimulation (*p* < 0.05, LPS24h vs Control; LPS24h vs LPS12h). Glycerol 3-phosphate is an important component of glycerophospholipid metabolism and is also an intermediate in the glycolysis metabolic pathway. It was highly significantly up-regulated in the cells after LPS stimulation (*p* < 0.01, LPS12h vs Control, LPS24h vs Control, LPS24h vs LPS12h). In a recent study of LPS-induced macrophage inflammatory responses^13^, some metabolites representing glycolysis increased after LPS stimulation, suggesting a role of glycolysis in inflammation in both bMECs and macrophages. As for other metabolites, sn-glycerol-3-phosphoethanolamine and O-phosphoethanolamine were found to be up-regulated significantly in LPS12h and LPS24h (*p* < 0.01) when compared to Control. The above findings indicated that after bMECs were stimulated by LPS, PCs, LPCs and other metabolites related to glycerophospholipid metabolism, glycerolipid metabolism and glucose metabolism had played anti-inflammatory, pro-inflammatory, antioxidant and energy-providing roles to regulate the cells, suggesting important functions of these pathways in bovine mammary inflammation.

α-LA and LA belong to ω-3 and ω-6 polyunsaturated fatty acids (PUFA) respectively. Pro-inflammatory and bioactive eicosanoids can be derived from LA^14^. α-LA has been shown an anti-inflammatory effect^15^, studies showed that increasing α-LA in diets can reduce the expression of the pro-inflammatory cytokine TNF-α, and the secretion of IL-1^16^. In the present study, there were significant reductions in α-LA and LA in LPS12h (*p* < 0.05). PGG2 and 15-keto-PGE2 were upregulated highly significantly in LPS12h (*p* < 0.01), consistent with previous studies, which showed that eicosanoids such as PGs are sensitive indicators of inflammation^1^. PGG2 is a precursor of PGE2, which is one of the most classical lipid mediators derived from AA catalyzed by cyclooxygenases (COXs). It is involved in acute inflammation or inflammatory immune diseases through several mechanisms, enhancing cytokine signaling by regulation of gene expression^17^. 15-keto-PGE2 is one of the metabolites of PGE2, which has been reported to down-regulate the expression of pro-inflammatory cytokines, to increase antioxidative transcription factor and nuclear related factor-2 (NRF2), to enhance the reactivity of antioxidation response, and to up-regulate the expression of antioxidant-related genes^18^. PGG2 and 15-keto-PGE2 decreased significantly in LPS24h relative to LPS12h (*p* < 0.01), suggesting that the anti-inflammatory or pro-resolving pathways might play a dominant role 24 h after LPS stimulation as the cells are likely restoring homeostasis. In addition to producing pro-inflammatory mediators, LA and α-LA are also precursors of AA, EPA and DHA respectively, which can be derived into anti-inflammatory or pro-resolving mediators^19^. It has been shown that patients with moderate or severe asthma and chronic obstructive pneumonia had lower levels of 11-HETE^20^, consistent with the significantly lower values in LPS12h in our study (*p* < 0.05). These findings show that there were strong immune responses characterized by enhanced α-LA and LA metabolism pathways in bMECs after LPS stimulation.

Among other lipids, there were significant changes in cis-9-palmitoleic acid, L-carnitine, acetylcarnitine and oleic acid. Palmitoleic acid is a monounsaturated fatty acid that can affect inflammatory markers, fat formation and peroxisome proliferator-activated receptor (PPAR) metabolic pathways^21^. It has been reported as having certain therapeutic effects in diseases such as metabolic syndrome, diabetes, inflammation and obesity^22^, affecting cellular lipid metabolism^23^, and improving glucose balance in the body^24^. Another monounsaturated fatty acid that was found to be differentially produced in the present study is oleic acid. Oleic acid-rich olive oil has proven not only to promote wound healing, but also to be beneficial for cancer, autoimmune and inflammatory diseases. Dietary oleic acid-rich olive oil can eliminate pathogens by interfering with macrophages, lymphocytes and neutrophils^25^. Cis-9-palmitoleic acid and oleic acid were down-regulated in bMECs after LPS stimulation, indicating enhanced lipid metabolism. This might suggest metabolic consumptions of them in the cells to play anti-inflammatory effect. Carnitine is an important factor in the metabolism of fatty acids in mammals, transferring fatty acids for decomposition, and is often used as a dietary supplement and food antioxidant. L-carnitine was highly significantly increased (*p* < 0.01) in LPS12h and LPS24h in this study. However, Xi *et al*.^7^ reported that carnitine metabolism was down-regulated during mastitis. Acetylcarnitine, which is close related to carnitine, was significantly increased in LPS12h as well (*p* < 0.01). This metabolite has been shown to be effective against pain caused by peripheral neuropathy, the pathogenesis of which includes inflammation^26^. It has been proposed as a potential biomarkers of allergic asthma and it also plays an important role in the insulin resistance pathway^27^. These findings might indicate the role of these lipids in anti-inflammatory, antioxidant and energy-regulating functions in the response of bMECs to LPS. They are consistent with the results from an earlier study which indicated that the synthesis of protein and fat in bMECs could be affected by LPS^6^.

Oxygen free-radicals and lipid peroxides can cause oxidative damage to cells. In the present study, the antioxidant pathways such as ascorbate metabolism and glutathione metabolism were affected significantly in bMECs after LPS stimulation. Myo-inositol participates in the process of biosynthesis of ascorbic acid, which functions as an antioxidant and is also very important for immune system. It was significantly up-regulated in LPS12h (*p* < 0.05) and LPS24h (*p* < 0.01) compared to Control, which is consistent with a study of RAW 264.7 showing a significant increase of myo-inositol after LPS stimulation^28^. UDP-D-glucuronate, also involved in ascorbate metabolism, decreased in LPS24h when compared to LPS12h (*p* < 0.05), as did one of its substrates - UPD-D-galactose. Glutathione, a classical antioxidant with antioxidative stress and detoxification effects which helps maintain the normal function of the immune system^29^, increased highly significantly in the cells in LPS12h (*p* < 0.01). Glutathione disulfide, a cofactor of glutathione peroxidase^30^ that has been reported to be involved with glutathione in the biosynthesis of leukotrienes^31^, was highly significantly up-regulated in LPS12h (*p* < 0.01). However, a study on LPS-stimulated macrophages showed that glutathione was the metabolite that decreased most after 6 h LPS stimulation, and the ratio of glutathione and glutathione disulfide also decreased significantly^13^. In the present study, there were significant increases (*p* < 0.05, LPS12h vs Control) in L-glutamate and L-glutamine, which are related to glutathione metabolism. Alanine, aspartate, and glutamate metabolism were all important in LPS-stimulated bMECs, which is consistent with the study of RAW 264.7 cells^28^, showing this metabolic pathway changed significantly in response to LPS. L-tyrosine was down-regulated in LPS12h and LPS24h, which is in agreement with the observation of the study of Xi *et al*.^7^, showing that milk tyrosine metabolism was altered in mastitis. These findings indicated that the metabolic pathways associated with antioxidants can be rapidly activated in bMECs after LPS stimulation, potentially producing antioxidants to remove excess oxygen free radicals, restoring normal operation of immune system, and maintaining or restoring the redox balance in bMECs during LPS-induced inflammation.

Amino acids are not only essential for protein synthesis in cell metabolism, but also act as intermediate metabolites that promote other biosynthetic reactions. It has been found that amino acid biosynthetic pathways are activated in cancer, and that specific amino acids are produced as important biomolecules such as the intermediates of nucleotides, lipids and glutathione^32^. Creatine is an amino acid derivative that is necessary for the rapid synthesis of ATP to supply energy. It is also a metabolite in the biosynthesis of arginine, an amino acid that has antioxidant properties^5,33^, as well as anti-inflammatory effects^34^. In the present study, creatine increased significantly in LPS12h (*p* < 0.05), which is consistent with LPS-stimulated RAW 264.7 cells^28^. L-aspartate and betaine have the ability to act against oxidation and inflammation^35,36^. Betaine decreased significantly in LPS24h compared to LPS12h (*p* < 0.05). L-aspartate decreased in LPS12h compared to Control (*p* < 0.05) and it reduced significantly in LPS24 compared to LPS12h (*p* < 0.01). Nicotinamide increased significantly in LPS12h (*p* < 0.05). Nicotinamide is the major source of NAD, is a key component of energy metabolism and signaling pathways. It is also an amide derivative of VB3, which has anti-inflammatory effect^37^. Nicotinamide can also inhibit the secretion of inflammatory cytokines and COXs-derived metabolites^38^. S-adenosylhomocysteine (SAH), one of key components in methionine cycle, increased significantly in macrophages after 6 h LPS stimulation^13^. In the present study, SAH significantly increased in LPS12h and LPS24h when compared to Control (*p* < 0.01). These results suggested that the energy metabolism of bMECs might be enhanced due to LPS stimulation, and metabolites with anti-inflammatory and antioxidant abilities could also be regulated through other pathways to play an important role in bMECs.

## Conclusion

In this study, an untargeted metabolomics method was used to analyze the expression pattern of metabolites in bMECs by LPS stimulation. 63 significant differential metabolites were identified. These differential metabolites were primarily involved in eight pathways including lipid metabolism and energy metabolism. The results suggested that bMECs can regulate pro-inflammatory, anti-inflammatory and antioxidant related metabolites to respond to inflammatory stimuli and promote cell homeostasis. Further studies are recommended using targeted metabolomics, lipidomics or proteomics to analyze the potential biomarkers and metabolic pathways identified in this trial, in order to provide screening biomarkers and therapeutic targets for bovine mammary gland inflammation.

## Methods

### Materials and study design

The cells used in this experiment were the 4^th^ passage of primary bovine mammary epithelial cells stored in the laboratory, which were isolated from healthy multiparous lactating Holstein cows using a procedure described previously^39^, and were purified and identified before storage. The purity of the cells was assessed at >98%. The cells are in round or elliptical, paving-stone shaped, have rapid proliferation and normal secretion function. The cells were identified by anti-cytokeratin 18 antibody and anti-vimentin antibody purchased from Bioss Biotechnology Co., Ltd (Beijing, China) (Supplementary Figs. S3 and S4). The cells were incubated with DMEM/F12 HEPES supplemented with 10% fetal calf serum after resuscitation. In experiments, they were randomly divided into 3 groups, with 6 biological replicates in each. Then challenged with 500 ng/mL LPS, samples were taken at 0 h, 12 h and 24 h post stimulation, named as Control, LPS12h and LPS24h respectively. In the pilot study, primers were designed with Primer 6.0 and synthesized by Shanghai Sangon Biological Engineering Technology & Services Co. Ltd (Shanghai, China) (Supplementary Table S4). Kits and reagents for all procedures from RNA extraction to RT-qPCR were purchased from TIANGEN Biotech Co., Ltd (Beijing, China) and TakaRa Co. Ltd (Dalian, China). Protein concentration was determined using ELISA assay kits. All the procedures were performed in accordance with the instructions provided by the companies. HPLC-Q-TOF MS detection technology was used in combination with data-dependent acquisition analysis methods to identify differential metabolites from these groups.

### Sample preparation and pretreatment

BMECs were washed twice with PBS after removal of the medium, then digested with trypsin and collected in a volume of 1 × 10^7^ cells per sample. After collection, the cells were washed with pre-cooled PBS, and spun at 1,000 r/min for 5 min at 4 °C, washed three times and removed the supernatant. To each sample, 1 mL of methanol: acetonitrile: distilled water (2: 2: 1, v / v) mixture was added to precipitate protein, pulverized by ultrasonic wave at low temperature for 30 min and then allowed them stand at −20 °C for 1 h. After that, samples were centrifuged at 13,000 r/min for 20 min at 4 °C, stored at −80 °C after removing the supernatant and vacuum drying. When dry, 100 μL of acetonitrile aqueous solution (acetonitrile: water = 1:1, v/v) was added and remixed, following brief vortexing, centrifugation was performed at 14,000 r/min for 15 min at 4 °C, and the supernatant was taken for experiment and analysis.

### HPLC-Q-TOF MS analysis

HPLC-Q-TOF MS analysis was performed by Shanghai Applied Protein Technology Co., Ltd. (Shanghai, China) using a 1290 Infinity Ultra-high Performance Liquid Chromatography system (Agilent Technologies, Palo Alto, CA, USA) coupled with a Triple TOF 5600 system (AB/SCIEX, Framingham, MA, USA).

Chromatographic separation was performed on a Waters ACQUITY UPLC BEH Amide 1.7 μm 2.1 mm × 100 mm column, and the injection volume was 2 μL. The column temperature was set at 25 °C and the flow rate was 0.3 mL/min. The mobile phase consisted of A: water + 25 mM ammonium acetate + 25 mM ammonia, and B (acetonitrile). The metabolites were eluted with a gradient of 95% B for 0–1 min; 95% to 65% B for 1–14 min; 65% to 40% B for 14–16 min; maintained at 40% B for 16–18 min; 40% to 95% B for 18–18.1 min; and then maintained at 95% B for 18.1–23 min. During the analysis, samples were placed in 4 °C auto-sampler, and in order to avoid the influence of the instrument detection signal fluctuations, continuous analysis of the samples were performed in a random order. The QC samples were inserted into the samples to monitor system stability and data quality as well.

For MS experiments, this study was conducted using electrospray ionization (ESI) positive and negative ion modes. The ESI source conditions after chromatographic separation were as follows: Ion Source Gas1 (Gas1): 60 psi, Ion Source Gas2 (Gas2): 60 psi, Curtain gas (CUR): 30 psi, source temperature: 600 °C, Ion Sapary Voltage Floating (ISVF) ± 5500 V (±ESI); TOF MS scan m/z range: 60–1000 Da, product ion scan m/z range: 25–1000 Da, TOF MS scan accumulation time 0.20 s/spectra, product ion scan accumulation time 0.05 s/spectra; the secondary mass spectra were acquired using information dependent acquisition (IDA) and used the peak intensity screening mode, Declustering potential (DP): ±60 V (±ESI), collision Energy: 35 ± 15 eV, IDA was set to dynamically exclude isotope ions within 4 Da and collect 6 fragment maps per scan.

### Data processing and statistical analyses

The data were converted into.mzXML format using ProteoWizard software (Version 3.0, Palo Alto, CA, USA)^40^, peak alignment, retention time correction and peak area extraction were performed using XCMS program^41^. Checked the integrity of the data obtained, deleted or supplemented the missing values and deleted the extreme values or ion peaks of which missed values more than 50%. The data of samples and metabolites were normalized to ensure that samples could be compared in parallel.

SIMCA-P (Version 14.1, Umetrics, Umea, Sweden) was used for multivariate statistical analysis. After pareto-scaling, unsupervised PCA was used to analyze the data, including QC samples consisting of 30 μL per sample, which can be used to detect the status of the instruments, balance the system and evaluate the stability. Tightly clustered QC samples can indicate the repeatability of the test and ensure that the stability of the instrumental analysis system is good, that the data are reliable, so that the differences in the metabolic profiles truly reflect the biological differences among the samples. Subsequently all samples were subjected to PLS-DA and OPLS-DA. Unlike PCA, PLS-DA is a supervised statistical method for discriminant analyses. OPLS-DA is a method that is calibrated on the basis of PLS-DA, filtering out noise that has nothing to do with the information classification, and improving the model’s analytical ability and effectiveness. R2Y and Q2 in OPLS-DA represent the rate of model interpretation and model predictive ability respectively, when 1 ≥ R2Y and Q2 ≥ 0.4, it indicates that the model is determined to be stable and reliable^42^. The VIP values obtained from OPLS-DA are used to measure the influence and interpretation ability of each metabolite expression pattern on the classification discrimination of each group, thereby assisting in the selection of marker metabolites. Finally, *t* - test and fold change analysis (calculate the average levels of a metabolite in one group relative to the other) were performed.

Potential biomarker screening was performed based on both OPLS-DA VIP > 1 and *p* < 0.05, and were identified by searching an in-house standard MS/MS library and online databases METLIN (http://metlin.scripps.edu) and HMDB (http://www.hmdb.ca) (using exact mass data (mass error ≤ 25 ppm) or MS/MS spectra matching)^41^. The in-house library contains MS/MS spectra of approximately 800 compounds which were obtained from standards. The MS/MS spectra that could not be matched to any of those in the in-house library were searched in online databases. Finally, MetaboAnalyst 4.0 (http://www.metaboanalyst.ca) and KEGG (http://www.genome.jp/kegg) were processed to cluster analysis and metabolic pathway analysis on differential metabolites.

## Supplementary information

Supplementary information

## Data Availability

The data used to support the findings of this study are available from the corresponding author by request.
