# Supplementary material for: Metabolomic Profiles of Bovine Mammary Epithelial Cells Stimulated by Lipopolysaccharide
Source: Sci Rep. 2019 Dec 13;9:19131. doi: 10.1038/s41598-019-55556-2 (PMC6911109; doi:10.1038/s41598-019-55556-2)
Supplement: Supplementary file 1 — Supplementary information [file 41598_2019_55556_MOESM1_ESM.pdf]

Metabolomic Profiles of Bovine Mammary Epithelial Cells Stimulated by Lipopolysaccharide

Y. X. Huang[1,2,3,*], L. H. Shen[1,2,*], J. Jiang[1,2], Q. P. Xu[1,2], Z. Z. Luo[1,2], Q. Luo[1,2], S. M. Yu[1,2], X. P. Yao[1,2], Z. H. Ren[1,2], Y. C. Hu[1,2], Y. X. Yang[4], and S. Z. Cao[1,2,+]

[1] Department of Clinical Veterinary Medicine, College of Veterinary Medicine, Sichuan Agricultural University, Chengdu, 611130, China

[2] Sichuan Provincial Key Laboratory of Animal Diseases and Human Health, Chengdu, 611130, China

[3] Institute of Biodiversity Animal Health & Comparative Medicine, College of Medical, Veterinary & Life Sciences, University of Glasgow, Glasgow, G61 1QH, UK

[4] Institute of Animal Science and Veterinary Medicine, Anhui Academy of Agricultural Sciences, Hefei, 230031, China

[+] Correspondence and requests for materials should be addressed to S. Z. Cao (email: suizhongcao@126.com)

* These authors contributed equally to this work

Supplementary Information

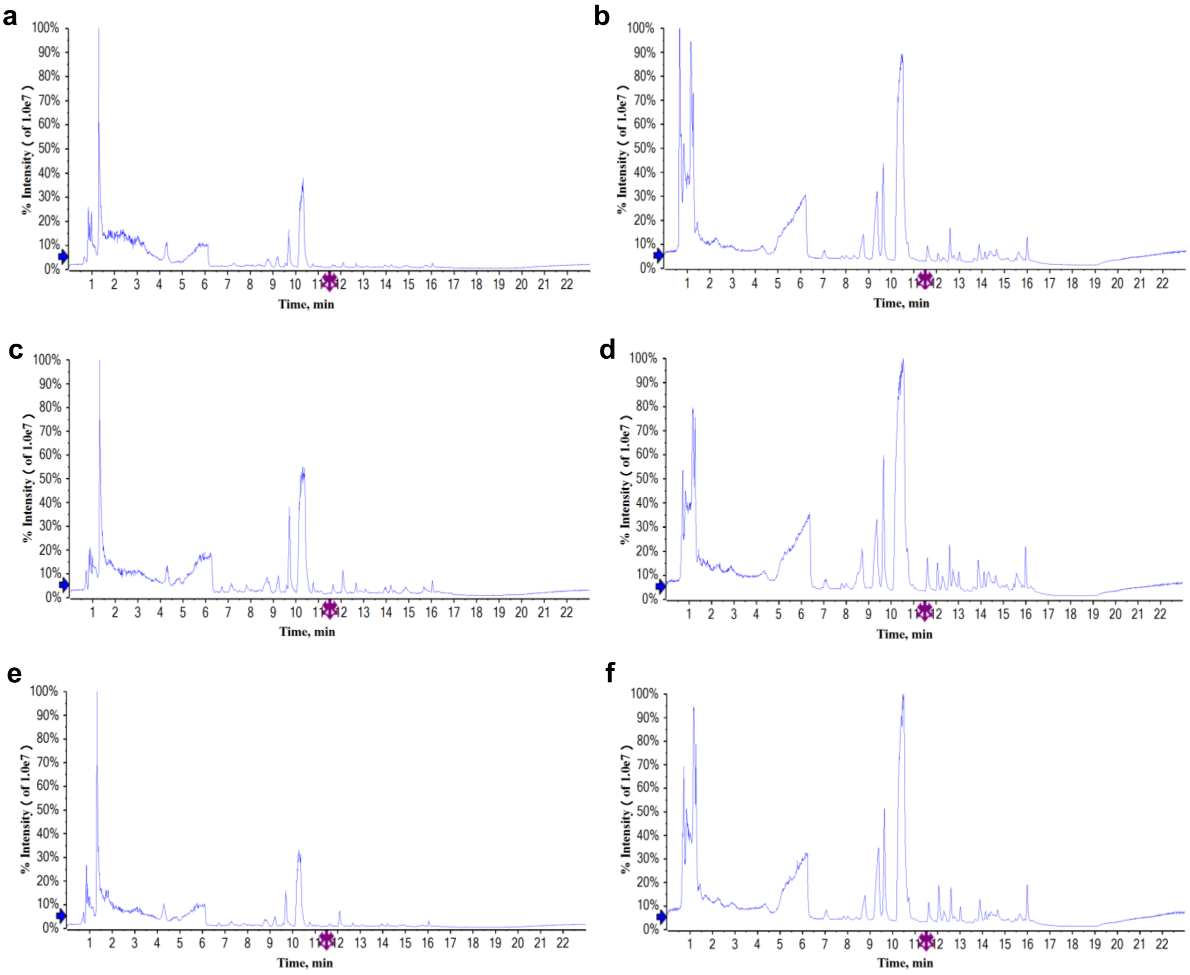

Fig. S1. Representative HPLC-Q-TOF MS Chromatograms. (a) and **(b)** are representative chromatograms of Control in ESI+ and ESI-, respectively. **(c)** and **(d)** are representative chromatograms of LPS12h in ESI+ and ESI-, respectively. **(e)** and **(f)** are representative chromatograms of LPS24h in ESI+ and ESI-, respectively.

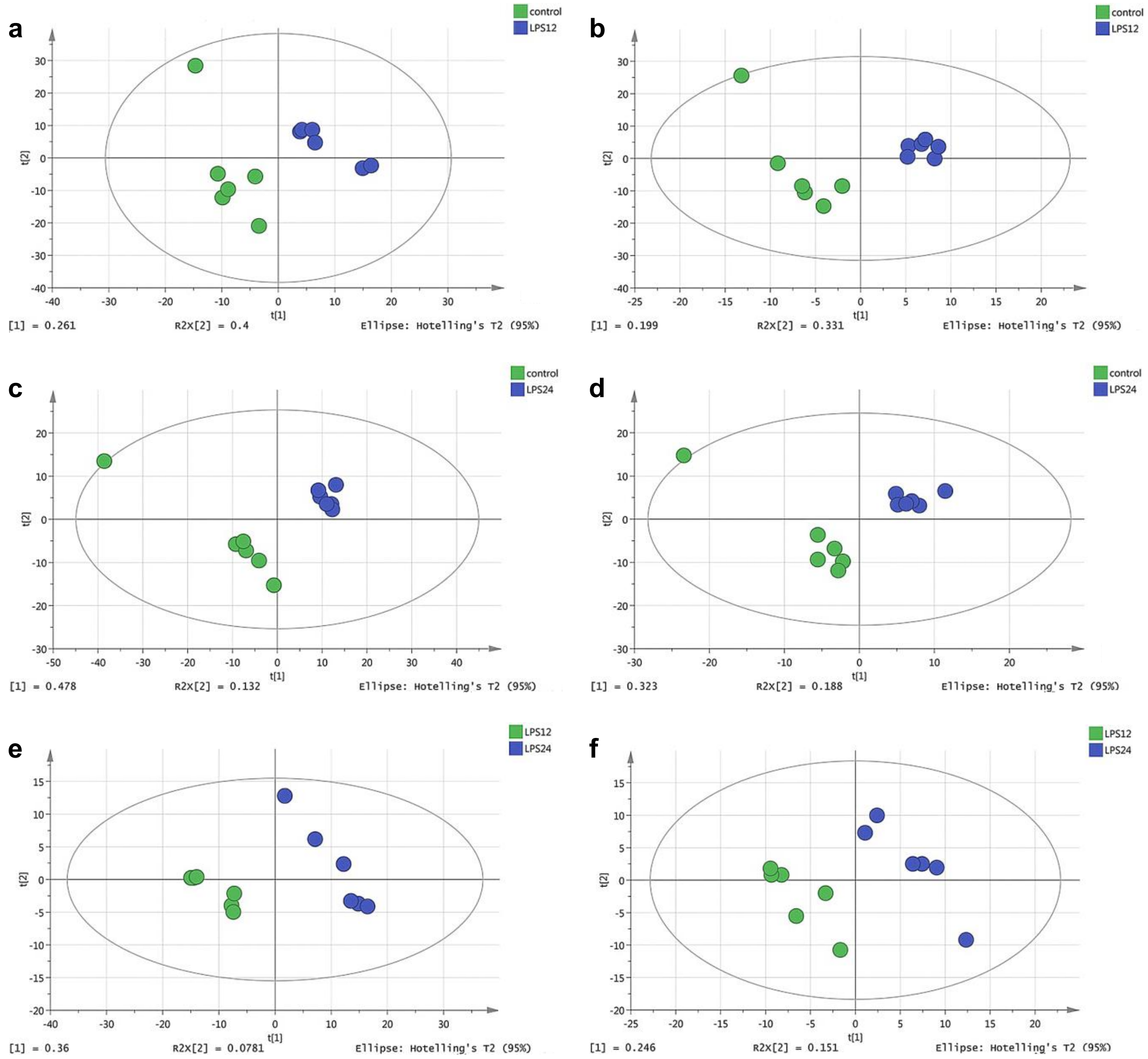

Fig. S2. PLS-DA scores plot of all groups. (a) and **(b)** are the PLS-DA score plots of LPS12h and Control in ESI+ and ESI-, respectively. **(c)** and **(d)** are the PLS-DA score plots of LPS24h and Control in ESI+ and ESI-, respectively. **(e)** and **(f)** are the PLS-DA score plots of LPS24h and LPS12h in in ESI+ and ESI-, respectively.

No.	Differential metabolites	m/z	rt	VIP	Fold change	*P*	Mode
1	PGG2	403.19	74.03	1.56	3.81	<0.01	ESI+
2	15-keto-PGE2	385.18	75.09	1.38	3.36	<0.01	ESI+
3	Glutathione	308.09	774.45	4.38	3.33	<0.01	ESI-
4	SAH	383.11	723.36	1.56	2.91	<0.01	ESI-
5	Acetylcarnitine	204.12	547.35	2.69	2.39	0.01	ESI+
6	Allocystathionine	221.06	864.43	1.77	2.39	<0.01	ESI-
7	L-Glutamate	189.09	743.26	1.09	2.38	0.01	ESI+
8	AMP	330.06	521.37	1.34	2.35	0.01	ESI+
9	L-Palmitoylcarnitine	400.34	313.71	2.91	2.29	<0.01	ESI+
10	L-Glutamine	188.1	658.39	1.17	2.17	0.03	ESI+
11	D-Fructose 1,6-bisphosphate	338.99	974.36	3.65	2.01	0.03	ESI-
12	L-Carnitine	162.11	659.17	2.8	1.91	<0.01	ESI+
13	cAMP	328.05	523.91	2.18	1.84	0.02	ESI-
14	S-Methyl-5'-thioadenosine	593.17	102	1.19	1.8	0.04	ESI-
15	sn-Glycerol-3-phosphoethanolamine	216.06	743.8	1.26	1.76	<0.01	ESI-
16	UDP	405.01	861.31	1.36	1.72	0.02	ESI+
17	PC (18:1(9Z)/18:1(9Z))	785.59	82.75	2.63	1.67	0.03	ESI+
18	Myo-Inositol	179.06	737.96	4.93	1.65	0.01	ESI-
19	NAD	664.11	864.01	1.04	1.6	0.05	ESI+
20	Nicotinamide	123.05	106.34	2.97	1.59	0.01	ESI+
21	Glycerophosphocholine	258.11	726.35	7.42	1.57	<0.01	ESI+
22	Glycerol 3-phosphate	171.01	725.41	1.71	1.54	<0.01	ESI-
23	Adenine	136.06	141.58	1.78	1.28	0.03	ESI+
24	Phosphorylcholine	184.07	891.65	4.68	1.25	0.01	ESI+
25	Glutathione disulfide	611.15	960.61	5.16	1.21	<0.01	ESI-
26	Creatine	132.08	647.98	4.04	1.18	0.03	ESI+
27	11-HETE	301.22	69.61	1.15	0.83	0.04	ESI+
28	cis-9-Palmitoleic acid	253.22	71.9	2.5	0.78	0.01	ESI-
29	LA	279.23	70.36	4.34	0.77	0.03	ESI-
30	α-LA	277.22	71.49	3.39	0.75	0.01	ESI-
31	L-Aspartate	132.03	768.64	2.49	0.73	0.05	ESI-
32	L-Tyrosine	180.07	541.43	2.36	0.69	0.05	ESI-
33	L-Valine	116.07	541.51	2.87	0.63	0.03	ESI-
34	9,10-DHOME	295.23	76.04	1.38	0.56	0.01	ESI+
35	2,3-dinor-TXB2	341.2	44.05	1.62	0.41	<0.01	ESI+
36	Uracil	111.02	143.94	2.42	0.39	<0.01	ESI-
37	Pregnenolone sulfate	395.19	44.3	3.2	0.37	0.01	ESI-
38	Progesterone	315.23	54.99	1.76	0.21	<0.01	ESI+

Tab. S1. Differential metabolites between LPS12h and Control. Ordered by fold change from larger to smaller. Fold changes are calculated as the average levels in LPS12h relative to those in Control, Fold change >1 indicates up-regulated, Fold change <1 indicates down-regulated.

No.	Differential metabolites	m/z	rt	VIP	Fold change	*P*	Mode
1	L-Palmitoylcarnitine	400.34	313.71	2.9	2.96	<0.01	ESI+
2	Phosphorylcholine	242.08	725.2	5.74	2.76	<0.01	ESI-
3	O-Phosphoethanolamine	140.01	875.76	2.47	2.72	<0.01	ESI-
4	SAH	383.11	723.36	1.4	2.53	0.01	ESI-
5	Glycerophosphocholine	258.11	726.35	7.87	2.1	<0.01	ESI+
6	sn-Glycerol 3-phosphoethanolamine	216.06	743.8	1.14	2.07	<0.01	ESI-
7	Glycerol 3-phosphate	171.01	725.41	2.51	2.05	<0.01	ESI-
8	L-Carnitine	162.11	659.17	2.17	1.87	<0.01	ESI+
9	PC (16:0/16:0)	756.55	193.78	5.59	1.81	0.03	ESI+
10	Myo-Inositol	179.06	737.96	5.46	1.72	<0.01	ESI-
11	Erucamide	338.34	55.1	1.65	1.7	0.03	ESI+
12	1-Stearoyl-sn-glycerol 3-phosphocholine	524.37	345.37	4.44	1.7	0.02	ESI+
13	Cholesterol sulfate	465.3	40.62	3.23	1.56	0.01	ESI-
14	α-LA	277.22	71.49	2.49	0.8	0.04	ESI-
15	Oleic acid	281.25	69.56	4.78	0.77	0.05	ESI-
16	L-Malic acid	133.01	775.16	2.22	0.68	0.04	ESI-
17	Succinate	117.02	742.47	1.89	0.67	0.01	ESI-
18	9,10-DHOME	295.23	76.04	1.03	0.64	0.05	ESI+
19	L-Tyrosine	180.07	541.43	2.27	0.64	0.05	ESI-
20	Uridine	243.06	286.5	1.78	0.63	0.04	ESI-
21	L-Aspartate	132.03	768.64	2.88	0.61	0.01	ESI-
22	UTP	482.96	961.25	1.93	0.59	0.03	ESI-
23	DL-2-Aminoadipic acid	160.06	780.25	1.35	0.58	0.02	ESI-
24	L-Valine	116.07	541.51	2.86	0.57	0.02	ESI-
25	UDP	403	906.97	2.84	0.54	0.05	ESI-
26	CTP	481.98	973.44	1.13	0.48	0.05	ESI-
27	Pyridoxine	170.08	185.06	2	0.43	0.03	ESI+
28	Uracil	111.02	143.94	2.26	0.41	<0.01	ESI-
29	CDP	402.01	932.02	1.82	0.36	0.04	ESI-
30	pregnenolone sulfate	395.19	44.3	3.18	0.34	<0.01	ESI-
31	Progesterone	315.23	54.99	1.52	0.14	<0.01	ESI+

Tab. S2. Differential metabolites between LPS24h and Control. Ordered by fold change from larger to smaller. Fold changes are calculated as the average levels in LPS24h to those in Control, Fold change >1 indicates up-regulated, Fold change <1 indicates down-regulated.

No.	Differential metabolites	m/z	rt	VIP	Fold change	*P*	Mode
1	SOPC	787.6	181.32	2.39	2.89	0.04	ESI+
2	PC (18:1(9Z)/18:1(9Z))	786.6	180.51	3.84	2.77	<0.01	ESI+
3	PC (16:0/16:0)	756.55	193.78	7.39	2.39	<0.01	ESI+
4	O-Phosphoethanolamine	140.01	875.76	2.36	1.85	0.01	ESI-
5	Glycerophosphocholine	258.11	726.35	5.57	1.33	0.05	ESI+
6	Glycerol 3-phosphate	171.01	725.41	2.02	1.33	<0.01	ESI-
7	sn-Glycerol 3-phosphoethanolamine	214.05	742.38	3.37	1.29	0.05	ESI-
8	L-Palmitoylcarnitine	400.34	313.71	1.58	1.29	0.02	ESI+
9	L-Aspartate	132.03	768.64	1.67	0.83	0.04	ESI-
10	Succinate	117.02	742.47	1.59	0.79	0.02	ESI-
11	Betaine	118.09	487.41	2	0.78	0.03	ESI+
12	Phosphorylcholine	184.07	891.65	4.11	0.72	0.01	ESI+
13	Allocystathionine	221.06	864.43	1.29	0.69	0.02	ESI-
14	UDP	403	906.97	2.55	0.68	0.02	ESI-
15	Adenine	136.06	141.58	1.57	0.68	<0.01	ESI+
16	DL-2-Aminoadipic acid	160.06	780.25	1.38	0.68	<0.01	ESI-
17	S-Methyl-5'-thioadenosine	356.1	104.42	5.71	0.65	0.02	ESI-
18	UDP-D-glucuronate	598.07	934.45	1.05	0.65	0.03	ESI+
19	CMP- sialic acid	613.14	852.09	1.45	0.64	<0.01	ESI-
20	D-Ribulose 1,5-bisphosphate	369	979.49	1.02	0.61	0.05	ESI-
21	L-Malic acid	133.01	775.16	3.34	0.61	0.01	ESI-
22	UDP-D-Galactose	584.09	861.19	1.19	0.6	0.01	ESI+
23	dGTP	505.99	939.55	8.79	0.55	0.02	ESI-
24	CDP	402.01	932.02	1.51	0.55	<0.01	ESI-
25	D-Fructose 1,6-bisphosphate	338.99	974.36	3.97	0.54	0.04	ESI-
26	ATP	508	944.42	3.57	0.54	0.01	ESI+
27	ADP	426.02	939.55	3.15	0.53	0.02	ESI-
28	cAMP	328.05	523.91	2.45	0.53	<0.01	ESI-
29	3-Phospho-D-glycerate	184.99	895.59	1.16	0.49	0.01	ESI-
30	UTP	482.96	961.25	3.09	0.49	0.01	ESI-
31	CTP	481.98	973.44	1.53	0.47	0.01	ESI-
32	AMP	330.06	521.37	1.24	0.35	<0.01	ESI+
33	15-keto-PGE2	385.18	75.09	1.56	0.28	<0.01	ESI+
34	PGG2	403.19	74.03	1.81	0.2	<0.01	ESI+
35	Phenylalanyl-Alanine	281.09	251.83	1.06	0.19	0.02	ESI+

Tab. S3. Differential metabolites between LPS24h and LPS12h. Ordered by fold change from larger to smaller. Fold changes are calculated as the average levels in LPS24h relative to those in LPS12h, Fold change >1 indicates up-regulated, Fold change <1 indicates down-regulated.

Gene	Primer sequence (F)	Primer sequence (R)	Length (bp)	GeneBank Number
GAPDH	5'- ACCCAGAAGACTGTGGATGG	3’-TTGAGCTCAGGGATGACCTT	125	NM_001034034
COX-2	5'- TTGTGTTCCCGTAGCCAAAT	3’-GAAATGATCTACCCGCCTCA	116	NM_174445.2
LOX-5	5'- GTTTGGCATCGCCATGTACC	3’-GCGTTGGCCTTGTCAAAGAG	147	NM_001192792.2
LOX-15	5'- CTCTTGGGTCCCTAACGC	3’-TGGAAGTTCGGCAGTGTT	99	NM_174501.2
TNF-α	5'- GGCAGACAGGATGTTGACCT	3’-TGACGGGCTTTACCTCATCT	133	NM_173966.3
IL-6	5'- AAGCAGCAAGGAGACAC	3’-GAACCCAGATTGGAAGC	82	NM_173923.2
CCL-2	5'- CCTGGGCAAGGAGTTAT	3’-TCAAGGCTTTGGAGTTT	94	NM_174006.2

Tab. S4. RT-qPCR primer sequence. Primers were designed with Primer 6.0 and synthesized by Shanghai Sangon Biological Engineering Technology & Services Co. Ltd (Shanghai, China).

a b

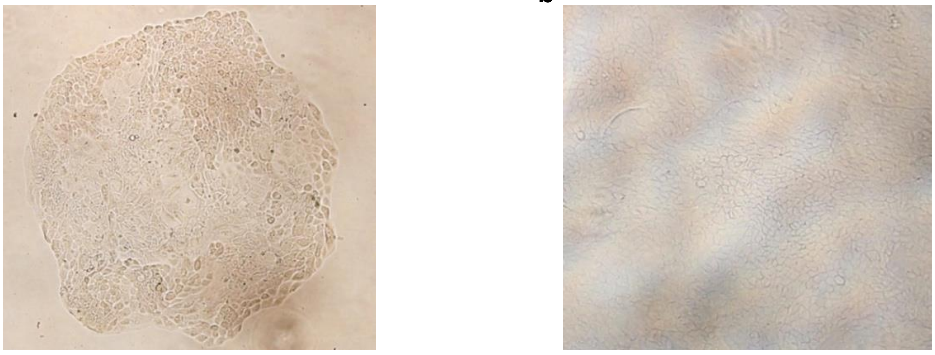

Fig. S3. Morphology of bMECs. (a) and **(b)** are purified bMECs (x 100). The cells are close to each other, form an island-shape. The cells are in round or elliptical, paving-stone shaped.

a b c d e f

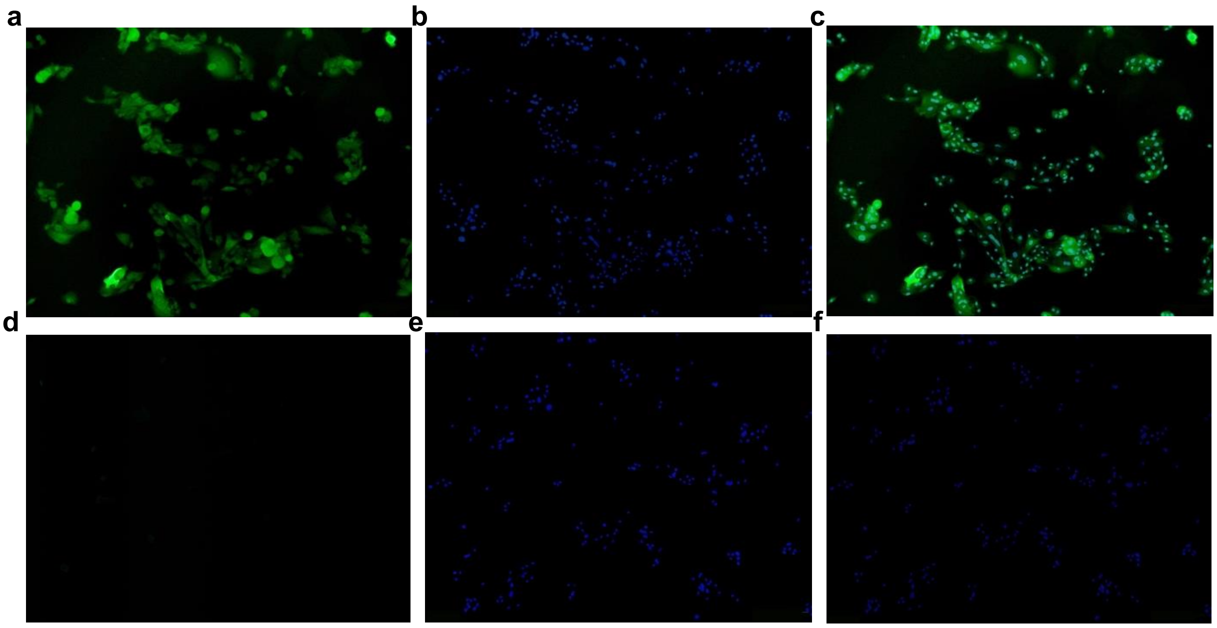

Fig. S4. Immunofluorescence staining of bMECs (× 400). (a) bMECs are positive for anti-cytokeratin 18 antibody. **(d)** bMECs are negative for anti-vimentin antibody. **(b)** and **(e)** are DAPI staining. **(c)** and **(f)** are combined by **(a)** and **(b)**, **(d)** and **(e)**, respectively.
